# *Clostridium perfringens* Beta2 toxin forms highly cation-selective channels in lipid bilayers

**DOI:** 10.1007/s00249-021-01577-7

**Published:** 2021-12-02

**Authors:** Roland Benz, Claudio Piselli, Cezarela Hoxha, Cornelia Koy, Michael O. Glocker, Michel R. Popoff

**Affiliations:** 1 Department of Life Sciences and Chemistry, Jacobs University, Campus Ring 1, 28759 Bremen, Germany; 2 Bacterial Toxins, Institut Pasteur, 28 Rue du Dr Roux, Paris, France; 3 Proteome Center Rostock, University Medicine Rostock, 18059 Rostock, Germany

**Keywords:** Pore-forming toxins, Consensus *Clostridium perfringens* Beta2 toxin (cCPB2), Propidium iodide uptake, Channel formation, Cation-selectivity, Lipid bilayer membrane

## Abstract

*Clostridium perfringens* is a potent producer of a variety of toxins. Well studied from these are five toxins (alpha, Beta (CPB), epsilon, iota and CPE) that are produced by seven toxinotype strains (A–G) of *C. perfringens*. Besides these toxins, *C. perfringens* produces also another toxin that causes necrotizing enterocolitis in piglets. This toxin termed consensus Beta2 toxin (cCPB2) has a molecular mass of 27,620 Da and shows only little homology to CPB and no one to the other toxins of *C. perfringens*. Its primary action on cells remained unknown to date. cCPB2 was heterogeneously expressed as fusion protein with GST in *Escherichia coli* and purified to homogeneity. Although cCPB2 does not exhibit the typical structure of beta-stranded pore-forming proteins and contains no indication for the presence of amphipathic alpha-helices we could demonstrate that cCPB2 is a pore-forming component with an extremely high activity in lipid bilayers. The channels have a single-channel conductance of about 700 pS in 1 M KCl and are highly cation-selective as judged from selectivity measurements in the presence of salt gradients. The high cation selectivity is caused by the presence of net negative charges in or near the channel that allowed an estimate of the channel size being about 1.4 nm wide. Our measurements suggest that the primary effect of cCPB2 is the formation of cation-selective channels followed by necrotic enteritis in humans and animals. We searched in databases for homologs of cCPB2 and constructed a cladogram representing the phylogenetic relationship to the next relatives of cCPB2.

## Introduction

*Clostridium perfringens* consensus Beta2 toxin (cCPB2) has been first identified as the unique virulence factor of a *C. perfringens* strain isolated from a piglet neonatal necrotic enteritis (Gibert et al. 1997). cCPB2 shows a low amino acid sequence identity (less than 15%) with *C. perfringens* Beta toxin (CPB) which is characteristic of *C. perfringens* types B and C and which is responsible for necrotic enteritis notably in young animals (Songer 2010; Uzal et al. 2010; Rood et al. 2018). *C. perfringens* harboring cCPB2 gene (*ccpb2*), CPB gene (*cpb*) alone, or both *cpb* + *ccpb2* were associated with necrotic enteritis in piglets, and strains containing *ccpb2* were also isolated from horses with typhlocolitis (Gibert et al. 1997). *C. perfringens* containing *ccpb2* were further described from a wide range of animals including piglets, horses, calves, chickens, dogs, and from humans (Herholz et al. 1999; Garmory et al., 2000; Manteca et al. 2002; Bueschel et al. 2003; Waters et al. 2003; Allaart et al. 2012; Lebrun et al. 2007; Carman et al. 2008; Ferrarezi et al. 2008; van Asten et al. 2010; Hazlett et al. 2011; Chan et al. 2012; Goldstein et al. 2012; Farzan et al. 2013; Gohari et al. 2014; França et al. 2016; Park and Rafii, 2019). *ccpb2* is localized on plasmid in *C. perfringens* and it is widespread among the *C. perfringens* strains (Gibert et al. 1997; Waters et al. 2003; Fisher et al. 2005; Harrison et al. 2005; Gurjar et al. 2010). Genetic investigations of *cpb2* in various *C. perfringens* strains from different origin showed a diversity of this toxin gene. *ccpb2*, is predominant in porcine isolates, whereas variants of *cpb2*, called atypical *cpb2* (*acpb2*), are harbored by non-porcine isolates (Jost et al. 2005). aCPB2 from the different *C. perfringens* types differ from 1 to 14 amino acid positions. A frameshift in *acpb2* gene in some *C. perfringens* strains results in the expression of a truncated CPB2 (Jost et al. 2005; Vilei et al. 2005). This might explain the absence of CPB2 effects in these strains. However, antibiotic treatment of *C. perfringens* with aminoglycosides such as gentamycin or streptomycin is able to induce a frameshifting and thus to allow the expression of *cpb2* as this has been demonstrated with *C. perfringens* strains from equine origin. Indeed, antibiotic treatment in horse predisposes to severe and fatal typhlocolitis with *C. perfringens cpb2* strains (Vilei et al. 2005). Albeit associations of *C. perfringens* harboring *cpb2* with intestinal diseases have been described, the role of cCPB2 and aCPB2 in pathogenesis remains unclear (van Asten et al. 2010; Schotte et al. 2004).

cCPB2 is a 27.6 kDa protein that has been found poorly cytotoxic in the intestinal cell line I407 (Gibert et al. 1997). No cytotoxic effect related to cCPB2 with *C. perfringens* isolates from porcine or human origin was observed in the porcine intestinal cell line IPI-21 or the human epithelial colorectal adenocarcinoma cells CaCo-2 (Allaart et al. 2014). Recently, cCPB2 was reported to induce apoptosis and inflammatory response in intestinal porcine epithelial (JPEC-J2) cells (Gao et al. 2020; Luo et al. 2020a). *C. perfringens* produces numerous protein toxins and *C. perfringens* strains are divided into seven toxinotypes (A–G) based on the combination of 6 toxins (alpha, CPB, epsilon (ETX), iota, enterotoxin (CPE), and NetB) (Rood et al. 2018). Despite a low level of amino sequence identity, most of *C. perfringens* toxins share a conserved structure of pore-forming toxins (PFT) (Popoff 2014). cCPB2 was crystallized, but the structure has not been determined yet (Gurjar et al. 2007).

Here, we report the pore-forming activity of cCPB2 in lipid bilayer membranes. Our results define this toxin as a cation-selective pore-forming component, which could be the primary cause of its action on target cells. Net negative charges control ion transport through channels formed by cCPB2. Their electrophysiological properties resemble conductance, selectivity, and size of the channels formed by *C. perfringens* enterotoxin (CPE) (Benz and Popoff 2018). We provide also a comparison of the pore-forming characteristics caused by the different toxins of *C. perfringens* and related bacteria in the discussion.

## Materials and methods

### Production and purification of consensus CPB2

*ccpb2* was PCR amplified from *C. perfringens* CWC245 (Waters et al. 2003) with the primers P2120 5’-GGATCCAAAGAAATCGACGCTTAT-3’ and P2121 5’-GTCGACCTATGCACAATACCCTTCACC-3’ adding *Bam*H1-*Sal*I and cloned into pET28a and pGEX4T1 yielding the recombinant plasmid pMRP1318 coding for GST-cCPB2. *Escherichia coli* BL21 harboring pMRP1318 was grown in LB medium (10 g/L tryptone, 5 g/L yeast extract, 10 g/L NaCl, pH 7.4) containing ampicillin (100 mg/mL) at 37 °C with shaking to an optical density at 600 nm of 0.7. The culture was induced with 1 mM isopropyl b-D thiogalactoside (IPTG) and incubated with shaking at 18 °C overnight. All media for cell growth were obtained from Gibco (ThermoFisher Scientific). After centrifugation, the pellet was suspended in phosphate-buffered saline (PBS) containing protease inhibitors (complete EDTA free protease inhibitors, Roche) and sonicated. The cell debris was separated from the soluble fraction by centrifugation (18,000 rpm, 15 min). The soluble fraction was applied onto a Glutathione Sepharose 4B column (GE Healthcare) equilibrated with PBS. The column was extensively washed with PBS and eluted with reduced glutathione 15 mg/mL in 50 mM TRIS–HCl pH 8. The fractions containing GST-cCPB2 were pooled, dialyzed against 50 mM TRIS–HCl, NaCl 150 mM, pH 7.5. Protein content was assayed with Coomassie brilliant blue G-250 (Bio-Rad protein assay). Cleavage by thrombin was performed with the Novagen thrombin kit (69,022–3) according to the manufacturer's recommendations.

### Mass spectrometry

Because of some inhomogeneity in the single-channel conductance histograms obtained for GST-cCPB2 and cCPB2 (see below), we subjected the GST-cCPB2 protein to mass spectrometry. The Coomassie-stained 1D-SDS gel band with GST-cCPB2 was worked up to generate tryptic peptides according to published protocols (Sinz et al. 2002; Konus et al. 2013; Röwer et al. 2018). Mass spectrometric analysis of peptide mixtures was performed in duplicate on a Synapt G2S mass spectrometer (Waters, Manchester, UK) using MassLynx version 4.1, coupled to a nanoACQUITY UPLC system (Waters MS-Technologies, Manchester, UK) via a NanoLockSpray ion source using a PicoTip Emitter (New Objective, Woburn, MA, USA) as described elsewhere (Sinz et al. 2002; Kumar et al. 2018). MS^E^ data were processed using ProteinLynx Global SERVER version 2.3 (Waters MS-Technologies). Protein identifications and partial amino acid sequence assignments were obtained by searching against all entries of a UniProt/Swiss-Prot (UniProt release 2021_01) database to which the sequence information of GST-cCPB2 was manually added (Röwer et al. 2009; Postu et al. 2019). Search parameters were set to: two missed cleavage sites, oxidation of methionine residues as variable modification, and carbamidomethylation of cysteines as fixed modification. Peptides were identified by at least three fragment ions. Singly charged peptide ions were rejected, whereas peptides with two, three, and four positive charges were accepted. Furthermore, peptides were removed from the hit list that had (1) a peptide score below 5.5, (2) a mass error above 13 ppm, and (3) less than six amino acid residues in length. Amino acid sequence coverage was graphically displayed on the GST-beta2-toxin fusion protein sequence.

### Lipid bilayer experiments

The methods used for the lipid bilayer measurements have been described previously in detail (Benz et al. 1978) from a 1% solution of diphytanoyl phosphatidylcholine, (DiPhPC; Avanti Polar Lipids, Alabaster, AL) in n-decane. The instrumentation consisted of a Teflon chamber with two aqueous compartments connected by a small circular hole with a surface area of about 0.4 mm^2^. The aqueous salt solutions (analytical grade; Merck, Darmstadt, Germany) were used unbuffered and had a pH around 6, because of the dissolved CO_2_ and the carbonic acid–bicarbonate equilibrium. Other pH-values are indicated. Recombinant GST-cCPB2 and cCPB2 cleaved with thrombin was added from concentrated stock solutions to the aqueous phase bathing membranes in the black state. The temperature was kept at 20 °C throughout. The conductance experiments were performed using a pair of Ag/AgCl electrodes with salt bridges switched in series with a voltage source and a highly sensitive current amplifier (Keithley 427, Keithley Instruments, Cleveland, Ohio). The amplified signal was monitored with a storage oscilloscope and the reconstitution of cCPB2 channels in the black lipid membrane was recorded with a strip chart recorder. Zero-current membrane potential measurements were obtained by establishing fivefold salt gradients across membranes containing about 100 cCPB2 channels as described elsewhere (Benz et al. 1985).

### Influence of net negatively charged groups of ion conductance of cCPB2 channels

The single-channel conductance of the cCPB2 channels was not a linear function of the bulk aqueous conductance (see “Results” section). One possibility to explain this effect represents a binding site inside the cCPB2 channel for cations or anions (Nelson and McQuarrie 1975; Benz and Hancock 1987; Trias and Benz 1993). Alternatively, it is also possible that charges at the pore mouth influence ion transport through cCPB2 channels. In such a case, the conductance depends on the square root of the bulk aqueous KCl concentration as it is shown in Table 1 (Trias and Benz 1993). The effect of point charges on channel conductance can be treated by a previously suggested formalism (Trias and Benz 1993). It assumes that the point charge is localized at the channel opening, where the negative charge *q*, causes a potential *Φ* on the mouth of a channel with a radius *r*, given by (Trias and Benz 1993):

**Table 1:** Average single-channel conductance of cCPB2 of *C. perfringens* in different electrolyte solutions

Electrolyte	Concentration (M)	G (pS)	*N*
KCl	0.01	53 ± 9	110
	0.03	94 ± 11	134
	0.1	219 ± 19	131
	0.15	243 ± 15	121
	0.3	350 ± 32	127
	1.0	642 ± 63	320
	3.0	1060 ± 95	149
LiCl	1.0	188 ± 24	105
KCH_3_COO (pH 7)	1.0	577 ± 63	196

cCPB2 was obtained by thrombin cleavage of GST-cCPB2. The membranes were formed from diphytanoyl phosphatidylcholine/ in *n*-decane. The single-channel conductance was measured at 20 mV applied voltage and *T* = 20 °C. N is the number of events used for the calculation of the average single-channel conductance G (± SD). Measurements in K-acetate were performed at pH 7, because of incomplete dissociation of potassium acetate at pH 6

1 $$\Phi =\frac{2q\cdot {e}^{-\frac{r}{{l}_{D}}}}{4\pi \cdot {\varepsilon }_{0}\cdot \varepsilon \cdot r},$$

${\varepsilon }_{0}$ (= 8.85 × 10^–12^ F/m) and ε (= 80) are the absolute dielectric constant of vacuum and the relative constant of water, respectively, and *l*_*D*_ is the so called Debye length, which controls the decay of the potential (and of the accumulated positively charged ions) in the aqueous phase (and also inside the channel:

2 $${{\varvec{l}}}_{{\varvec{D}}}^{2}=\frac{{\varvec{\varepsilon}}\cdot {{\varvec{\varepsilon}}}_{0}\cdot {\varvec{R}}\cdot {\varvec{T}}}{2\cdot {{\varvec{F}}}^{2}\cdot {\varvec{c}}},$$

*c* is the bulk aqueous salt concentration, and *R* is the gas constant, *T* the absolute temperature (in °K), and *F* the Faraday constant (*RT/F* = 25.2 mV at 20 °C). The concentration of the monovalent cations near the point charge increases because of the negative potential. Their concentration, ${c}_{0}^{+}$ at the channel mouth is given by:

3 $${{\varvec{c}}}_{0}^{+}={{\varvec{c}}}_{0}\cdot {{\varvec{e}}}^{-\frac{\boldsymbol{\Phi }\cdot {\varvec{F}}}{{\varvec{R}}\cdot {\varvec{T}}}.}$$

The cation concentration, ${c}_{0}^{+}$, at the mouth of the pore can now be used for the calculation of the effective conductance-concentration curve:

4 $${\varvec{G}}\left({\varvec{c}}\right)={{\varvec{G}}}_{0}\cdot {{\varvec{c}}}_{0}^{+}.$$

${G}_{0}$ is the concentration independent conductance of the channel.

### Cell culture

Vero, MDCK, HUVEC cells were grown in Dulbecco's modified Eagle medium (DMEM) + Glutamax™ (Gibco, Thermo Fisher Scientific) supplemented with 10% fetal calf serum (Gibco) and containing 100 units/mL penicillin G sodium and 100 μg/mL streptomycin sulfate (Penicillin Streptomycin, Gibco) at 37 °C in a 5% CO_2_ incubator. For cytotoxicity and propidium iodide (PI) (Roche) assays, cells were grown to confluency in 96-well plates. The monolayers were washed once in DMEM and incubated in PBS containing 5 mM glucose, 0.1% BSA (Gibco) and serial dilutions of CPE in DMEM (100 μl final volume in each well).

### Propidium iodide (PI) influx

For assay of (propidium iodide) PI entry, HUVEC cells were grown on 96-well plates until confluency. PI (Roche) (5 μg/mL) was added in the culture medium together with toxins. The plates were read with a spectrofluorimeter (Fluoroskan II) (excitation 540 nm and emission 620 nm) at 1, 2, 4, and 22 h. The results were expressed as the percentage of fluorescence obtained by treatment with Triton X-100 (0.2%) for 30 min at 37 °C.

## Results

### Purification of GST-cCPB2 and cCPB2

**Fig. 1:**
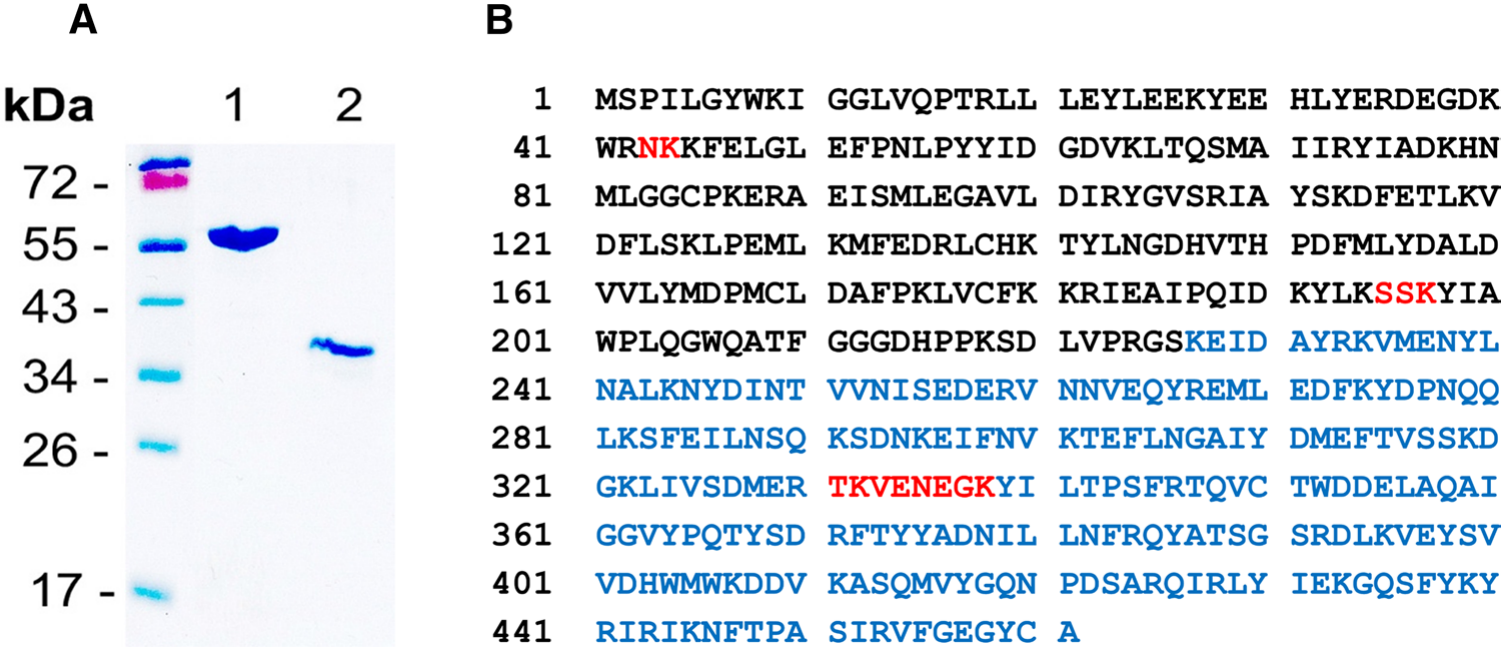
**A** 10% SDS–PAGE of purified consensus GST-CPB2, and consensus CPB2. Lane 1 shows 2 µg of recombinant consensus GST-CPB2 dissolved in 10 µL sample buffer according to Laemmli (1970); lane 2 µg thrombin cleaved GST-CPB2. The left lane of the SDS–PAGE shows the location of the molecular mass markers: 17, 26, 34, 43, 55, and 72 kDa. The gel was stained with Coomassie brilliant blue. **B** Graphical presentation of the amino acid sequence coverage after tryptic in-gel digestion of GST-cCPB2 excised from protein band in A, lane 1. Amino acid residues (single letter code) shown in black indicate the partial sequence of GST and those in blue that of cCPB2, which were represented by matching peptide ion signals. Amino acid residues shown in red point to partial sequences for which no matching peptide ion signals were observed by nanoLC-ESI-MS^E^ analysis. Amino acid position numberings are given at the left

Purified recombinant GST-cCPB2 and cCPB2 as obtained by thrombin cleavage of GST-cCPB2 were essentially free of contaminant proteins as shown in Fig. 1 by a 10% SDS–PAGE. To verify that the 55 kDa band in the SDS gel (Fig. 1A; lane 1) is indeed GST-cCPB2 and does not contain contaminant proteins, a 55 kDa band from a similar SDS–PAGE experiment was excised and subjected to in-gel trypsin treatment followed by peptide mass fingerprinting. Partial amino acid sequences of the GST-cCPB2 were matched to 209 peptide ion signals and resulted in a 96.7% amino acid sequence coverage (Fig. 1B), clearly identifying the protein. In addition, elongation factor Tu (45–50 kDa) from *E. coli* was found to be present as minor contamination in the band (data not shown). The mass spectrometry results verified that we were indeed working with GST-cCPB2 and upon thrombin cleavage with cCPB2, respectively, as the only pore-forming compounds in the protein extracts.

### Pore-forming activity of GST-cCPB2 and cCPB2 toxin in lipid bilayers

**Fig. 2:**
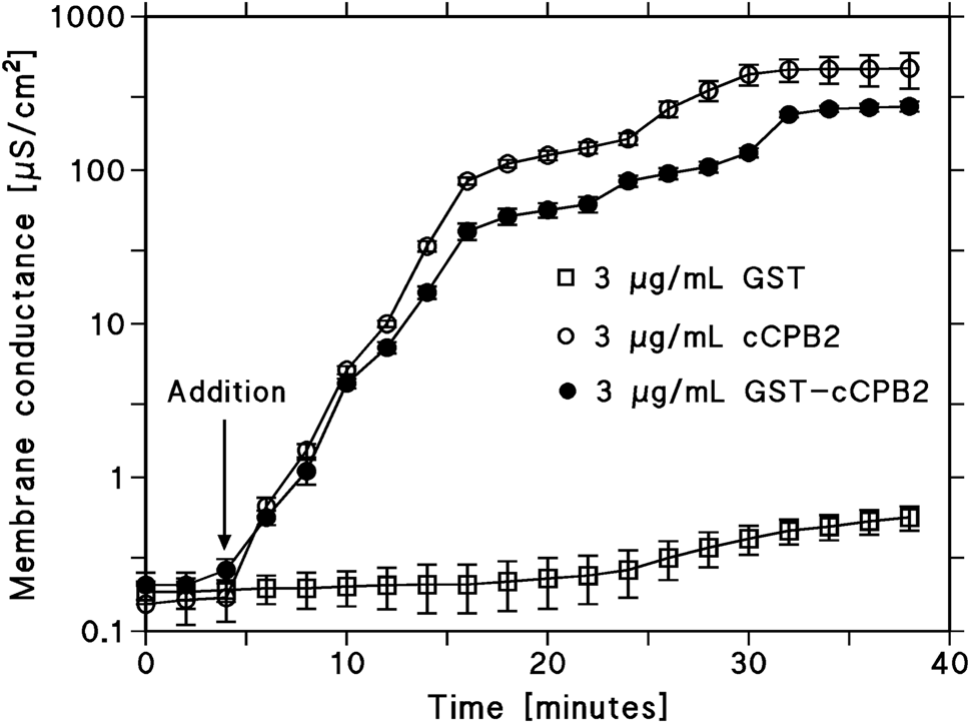
Specific membrane conductance as a function of time after the addition of GST, GST-cCPB2, and cCPB2 to membranes formed of DiPhPC/n-decane. The arrow indicates the addition of the three compounds in a concentration of 3 µg/mL to the aqueous phase bathing individual black bilayers. The data points show the mean ± SD of three experiments. The aqueous phase contained 1 M KCl, pH 6. The applied voltage was 20 mV; *T* = 20 °C

In a first set of experimental conditions, we studied the pore-forming ability of the recombinant GST-cCPB2 fusion product in lipid bilayer membranes made of DiPhPC/n-decane. Surprisingly, the addition of this construct in a concentration of about 3 µg/mL to black lipid bilayer membranes resulted in a strong increase of the membrane conductance within about 25 min (see Fig. 2). This result suggested that GST-cCPB2- represents a channel-forming component that could boost the conductance of artificial membranes. When we removed the GST-tag by treatment of GST-cCPB2- with thrombin and added cCPB2 in the same concentration to black lipid bilayer membranes, we observed a similar conductance increase. cCPB2 and GST-cCPB2- in a concentration of about 3 µg/mL had approximately the same effect on lipid membranes, i.e. the addition of both proteins resulted in a strong conductance increase from ground level conductance (about 10^–7^ S/cm^2^) to 10^–4^ S/cm^2^ within about half an hour as shown in Fig. 2. The addition of GST alone at the same concentration as the toxins did not result in any appreciable increase of membrane conductance as Fig. 2 clearly demonstrates.

### Single conductance of cCPB2 channels

**Fig. 3:**
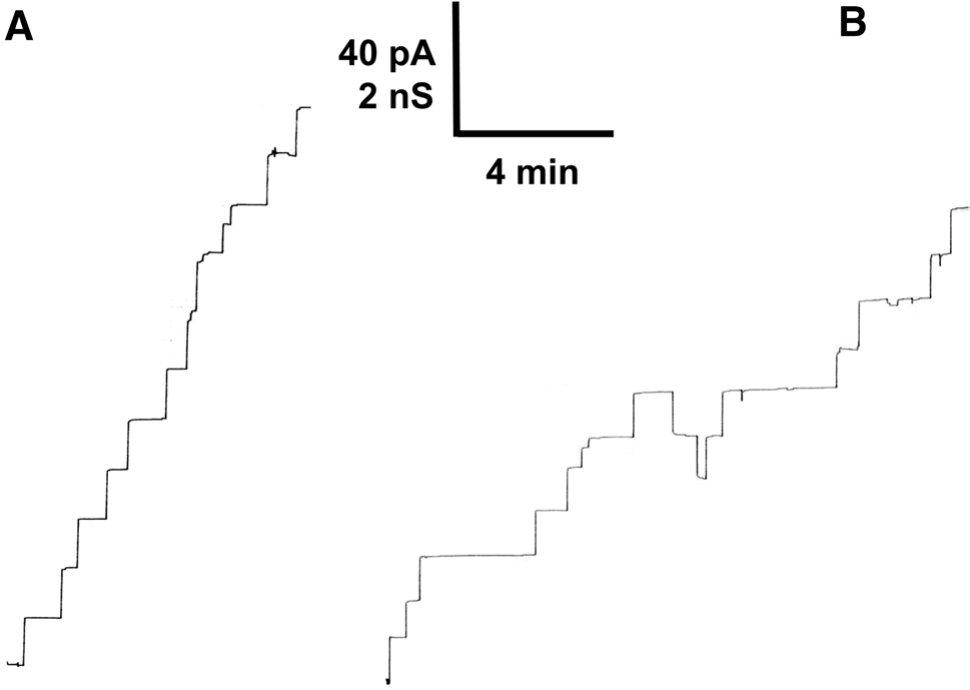
Single-channel recording of a DiPhPC/*n*-decane membrane in the presence of cCPB2 of *C*. *perfringens* cleaved with thrombin (**A**) and GST-cCPB2 (**B**). The aqueous phase contained unbuffered 1 M KCl, pH 6.0, and about 30 ng/mL cCPB2 (**A**) and about 40 ng/mL GST-cCPB2 (**B**). The applied membrane potential was 20 mV and the temperature was 20 °C. The recording was performed with the strip chart recorder at a time resolution of 10 Hz

The addition of cCPB2 in a much smaller concentration than added above in the multi-channel experiments allowed the resolution of conductance steps as demonstrated in Fig. 3. Shortly after the addition of 30 ng/mL cCPB2 to one side of a black DiPhPC/n-decane membrane the conductance increased in discrete steps that indicated the formation of ion-permeable channels in the membrane. Most of the conductance steps were directed upward and terminating events were only rarely observed. This observation suggested long lifetimes of the channels formed by cCPB2, similar to channels formed by other toxins and binding proteins of A–B toxins of *C. perfringens* (Schmid et al. 1994; Petit et al. 2001; Knapp and Benz 2016; Benz and Popoff 2018). Channel formation was frequent, which allowed meaningful statistics of channels formed by cCPB2 in lipid bilayer membranes. For comparison, we also included into Fig. 3 also a current recording, which we obtained in single-channel experiments with uncleaved GST-cCPB2 under identical conditions to the trace in Fig. 3A (1 M KCl, pH6). See the current trace in Fig. 3B.

**Fig. 4:**
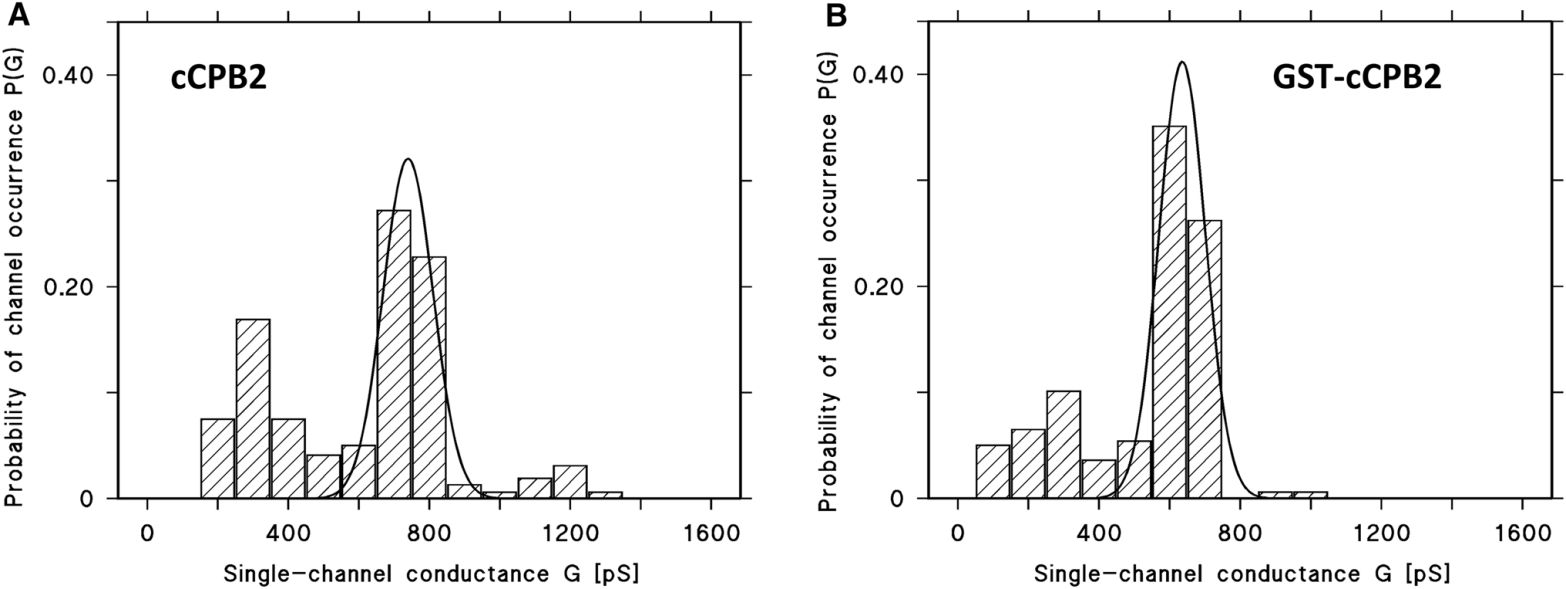
Histogram of the probability P(G) for the occurrence of a given conductivity unit in DiPhPC/*n*-decane membranes in the presence of cleaved cCPB2 and GST-cCPB2 of *C. perfringens*. The probability of channel occurrence was calculated by dividing the number of fluctuations with a given conductance unit by the total number of conductance fluctuations in the presence of cCPB2 and GST-cCPB2, respectively. **A** The mean value of the single-channel conductance of all conductance fluctuations was 643 pS for 320 single-channel events collected from different individual membranes. Aqueous phase contained 1 M KCl, pH6, and about 30 ng/mL cCPB2 cleaved with thrombin. The applied voltage was 20 mV and the temperature was 20 °C. The solid line shows a fit of the histogram with a Gaussian distribution. The maximum of the distribution is at a probability of 0.321 ± 0.082 and the conductance is 740 ± 71 pS for all single events taken from nine individual membranes. **B** The mean value of the single-channel conductance of all conductance fluctuations with GST-cCPB2 was 530 pS for 168 single-channel events collected from different individual membranes. The aqueous phase contained 1 M KCl, pH6, and about 40 ng/mL GST-cCPB2. The applied voltage was 20 mV and the temperature was 20 °C. The solid line shows a fit of the histogram with a Gaussian distribution. The maximum of the distribution is at a probability of 0.412 ± 0.061 and the conductance is 636 ± 66 pS for all single events taken from five individual membranes

Figure 4A shows a histogram of all conductance fluctuations observed with cCPB2 that was obtained by thrombin cleavage of GST-cCPB2. The distribution of cCPB2 channels showed two maxima. A minor fraction of about 25% of all fluctuations centered on 300 pS and the major one with more than 50% of the channels around 700–800 pS in 1 M KCl, pH 6. The mean value of all fluctuations was 642 ± 63 pS. We performed also single-channel conductance measurements with GST-cCPB2 because this construct showed also membrane activity in multi-channel experiments. The current recordings with this construct showed similar fluctuations to cCPB2 as shown in Fig. 3B. However, statistics of the single-channels formed with this compound suggested that the conductance of the channels shown in Fig. 4B was on average slightly smaller than that for cCPB2. This could mean that the GST-tag attached to cCPB2 had some minor influence on the channel conductance possible caused by sterical hindrance of ion flux although it did not influence its membrane activity.

### Single-channel analysis

Because of the slight difference between the single-channel conductance observed with GST-cCPB2 and cCPB2 cleaved with thrombin we performed all further experiment with cCPB2. In particular, we measured the single-channel conductance of cCPB2 channels for a variety of KCl-concentrations and for 1 M KCH_3_COO^−^ and 1 M LiCl. The experiments were performed similarly to those of Figs. 3 and 4. The results are listed in Table 1. For all conditions, the single-channel conductance showed a major maximum for about 50–70% of all conductance fluctuations. A minor peak of about 30–40% of all steps had a conductance that was about half of the major peak. The single-channel measurements under the different conditions were performed to see whether the single-channel conductance was a linear function of the bulk aqueous KCl concentration. Similarly, we exchanged in the salt solutions, mobile potassium ions and chloride by the less mobile Li^+^ and acetate ions to get some information about size and selectivity of the channels formed by cCPB2. The results of these measurements are summarized in Table 1. They clearly demonstrate that the single-channel conductance did not follow a linear function of the bulk aqueous concentration, which indicated that the movement of ions through the cCPB2 channels is controlled by net charges in or near the channels, i.e. they are highly selective. This can also be judged from the measurements in 1 M LiCl and 1 M KCH_3_COO^−^. The exchange of chloride by the less mobile acetate had an only minor effect on the single-channel conductance whereas the replacement of K^+^ by the less mobile Li^+^ changed substantially channel conductance indicating its cation selectivity. The measurements with potassium acetate were performed at pH 7 because of incomplete dissociation of KCH_3_COO^−^ at pH 6. Single-channel experiments at different pH-values between pH 5 and pH 8 showed that channel-forming activity and channel conductance did not change in this pH-range.

### Selectivity of the cCPB2 channel

To confirm the possible cation selectivity of the cCPB2 channel, we performed zero-current membrane potential measurement in the presence of fivefold salt gradients of KCl, LiCl, and KCH_3_COO^−^. The salt gradients were established when about 100–1000 cCPB2 channels were reconstituted into the lipid bilayers. Afterwards the instrumentation was switched to the measurement of zero-current membrane potentials. The potential turned in all experiments to positive values at the more dilute side (0.1 M) of the membranes containing cCPB2 channels. These results indicated again preferential movement of cations for all three salts. The analysis of the zero-current membrane potentials using the Goldman–Hodgkin–Katz equation (Benz et al. 1985) demonstrated indeed that cCPB2 formed highly cation-selective channels with permeability ratios for cations and anions that were around ten and higher for all three salts studied here (Table 2). Similar permeability ratios were also found for CPE (Benz and Popoff 2018) and Beta toxin (CPB) of *C. perfringens* (Shatursky et al. 2000), whereas ETX-channels were preferentially anion selective (Petit et al. 2001).

**Table 2:** Zero-current membrane potentials, *V*_m_, of DiphPC/*n*-decane membranes in the presence of *C. perfringens* consensus Beta2 toxin (cCPB2) measured for fivefold gradients of different salts^a^

Electrolyte	Permeability ratios *P*_cation_/*P*_anion_	*V*_m_ (mV)
KCl	11.8	33.0 ± 1.8
LiCl	9.8	30.9 ± 1.5
KCH_3_COO^−^ (pH 7)	15.0	34.5 ± 2.1

^a^*V*_m_ is defined as the difference between the potential at the dilute side minus the potential at the concentrated side. The aqueous salt solutions were used unbuffered and had a pH of 6, if not indicated otherwise; *T* = 20 °C. The permeability ratios *P*_cation_/*P*_anion_ were calculated from the zero-current potentials using the Goldman–Hodgkin–Katz equation (Benz et al. 1985). The zero-current potentials are given as the mean (± SD) of at least three individual experiments. Measurements in K-acetate were performed at pH 7, because of incomplete dissociation of potassium acetate at pH 6

### Cell cytotoxicity

**Fig. 5:**
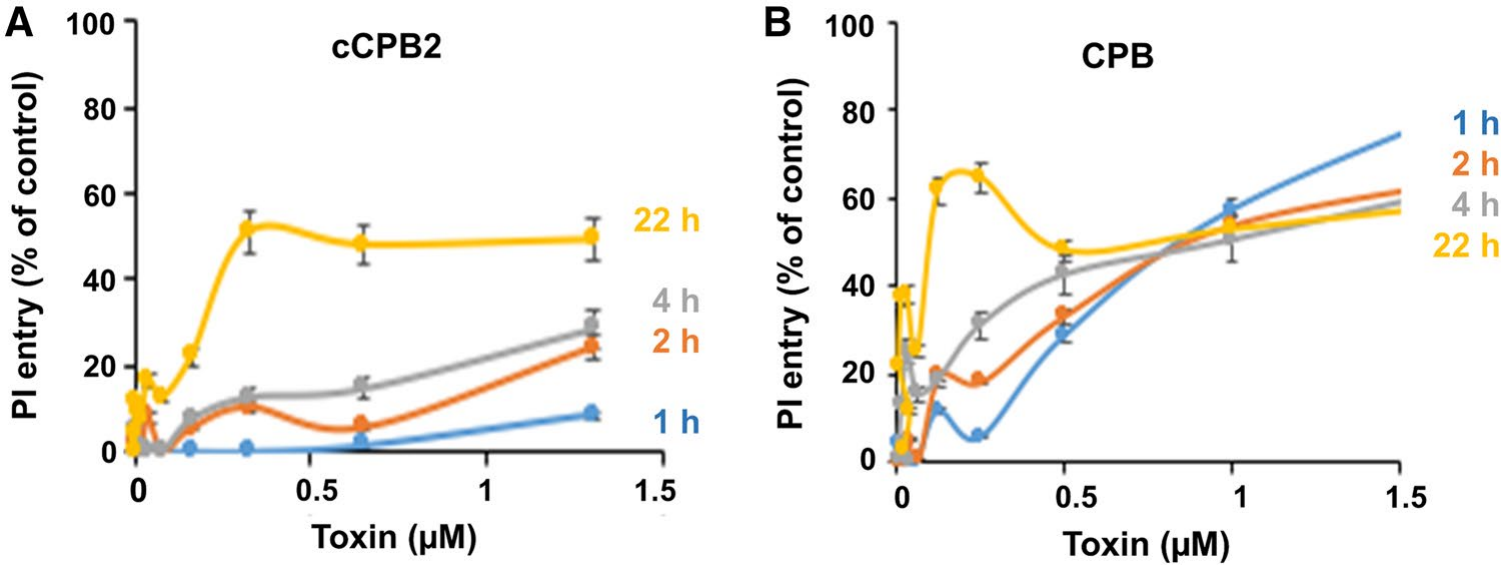
Cytotoxicity of GST-cCPB2 (**A**) and CPB (**B**) in HUVEC cells monitored by PI entry. HUVEC cells in DMEM medium containing 5 μg/mL were incubated with GST-cCPB2 (**A**) or CPB (**B**) for 1–22 h at 37 °C. PI entry was monitored by spectrofluorometry. The data are expressed as percentages of fluorescence compared to the control consisting of cells treated with 0.2% Triton X-100. Data were collected from three experiments and means and standard deviation were calculated

GST-cCPB2 and *C. perfringens* enterotoxin (CPB) were tested for cytotoxicity in human umbilical endothelial cells (HUVEC) by monitoring the entry of propidium iodide (PI) as already used for other *C. perfringens* pore-forming toxins such as *C. perfringens* enterotoxin (CPE) or *C. perfringens* epsilon toxin (Petit et al. 2003; Benz and Popoff 2018). CPB has been found to be specifically cytotoxic for endothelial cells (Gurtner et al. 2010; Popescu et al. 2011). Indeed, as shown in Fig. 5B, CBP efficiently promoted entry of PI into HUVEC cells. In contrast, cCPB2 showed a weak activity in HUVEC (see Fig. 5A). Only a prolonged incubation with cCPB2 to 22 h induced significant PI entry into cells, whereas CPB caused higher effects at earlier incubation times in the same range of toxin concentrations. No activity was observed with cCPB2 or CPB in intestinal or kidney epithelial cells (CaCo-2, Vero, MDCK cells) as monitored by PI entry or with viability test (WST-1).

## Discussion

### Consensus CPB2 is a channel-forming toxin

In contrast to CPB that is specifically cytotoxic for endothelial cells (Gurtner et al. 2010; Popescu et al. 2011), recombinant cCPB2 showed a weaker cytotoxicity in human endothelial cells (HUVEC), and no one in intestinal and kidney epithelial cells as tested in Vero, MDCK, and CaCo-2 cells. This does not preclude that cCPB2 and aCPB2 recognize specific cell type of cell subsets. Indeed, ETX is active in only a small number of kidney cell lines (Popoff 2011). Initially, cCPB2 was found to induce rounding of intestinal epithelial cells I407 (Gibert et al. 1997). More recently, cCPB2 was reported to be cytotoxic for intestinal porcine epithelial (JPEC-J2) cells and to disrupt the intercellular junctions (Gao et al. 2020; Luo et al. 2020a). The cCPB2-induced inflammatory response and apoptosis seems to be regulated by micro RNAs (Gao et al. 2021a, b; Luo et al. 2020b). Thereby, cCPB2 possibly promotes cytotoxic activity only in specific cell lines.

Nevertheless, it is clear from the data presented here that cCPB2 is a channel-forming toxin. This comes in particular from the high pore-forming activity of both GST-cCPB2 and cCPB2 obtained by cleavage with thrombin. Both proteins formed well-defined channels with a long lifetime, which is quite typical for channel-forming toxins. We observed channels with two different conductance values: a minor fraction had a conductance of about 300 pS in 1 M KCl, whereas more than 50% of all channels had a conductance around 700pS. Careful analysis of the current recordings suggested that the channel with the higher conductance was not caused by simultaneous insertion of two small channels or by impurities within cCPB2. It seems, moreover, that the formation of two types of channels is an intrinsic property of the pure cCPB2. We could speculate that the number of cCPB2 monomers in a channel-forming unit is responsible for the two types of channels. Many of the pore-forming toxins (Kitadokoro et al. 2011; Iacovache et al. 2016; Savva et al. 2019); and the binding components of the A–B toxins form channels with seven subunits (Barth et al. 2004; Schleberger et al. 2006; Jiang et al. 2015). Indeed, variability in channel conductance, likely due to different oligomer size (hexamer/heptamer), has been observed with CPB and *Staphylococcus aureus* alpha-hemolysin (Czajkowsky et al. 1998; Shatursky et al. 2000; Song et al. 1996). This could also be the case for cCPB2 because of its low homology to CPB. A change in the stoichiometry of the channel-forming complex to six monomers could well account for channels that have a smaller single-channel conductance.

### Beta-strands form presumably the consensus CPB2 channel

**Fig. 6:**
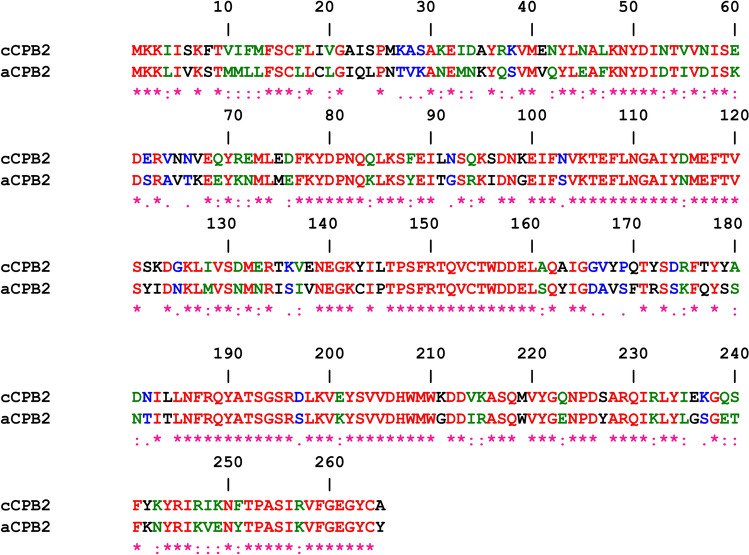
Amino acid sequence alignment of cCPB2 and aCPB2 of *C. perfringens*. The alignment was performed using the NCBI Reference protein sequences WP_096517132 and AAC27654, respectively, and Pole Bioinformatique Lyonnaise Network Protein Sequence Analysis (https://npsa.lyon.inserm.fr/cgi-bin/align_clustalw.pl). Amino acids identical in the two proteins are highlighted in red (*), strongly similar amino acids (:) are given in green and weakly similar ones (.) in blue. The mature amino acid sequence starts for both proteins with amino acid 31

Several versions of CPB2 are known. We performed here experiments with the plasmid-encoded cCPB2 (accession number AAC27654; (Gibert et al. 1997)). The other version of CPB2, also known as atypical CPB2 (WP_096517132; (Jost et al. 2005)) has identity to cCPB2 of 62.3% and similarity of 24.9%. Differences are 12.8%. Figure 6 shows an alignment of cCPB2 with aCPB2 using ClustalW multiple alignment (https://npsa.lyon.inserm.fr/cgi-bin/align_clustalw.pl). The alignment demonstrates the high homology between both toxins. There exists only little doubt that also atypical Beta2 is a channel-forming component because of its high homology to cCPB2.

**Fig. 7:**
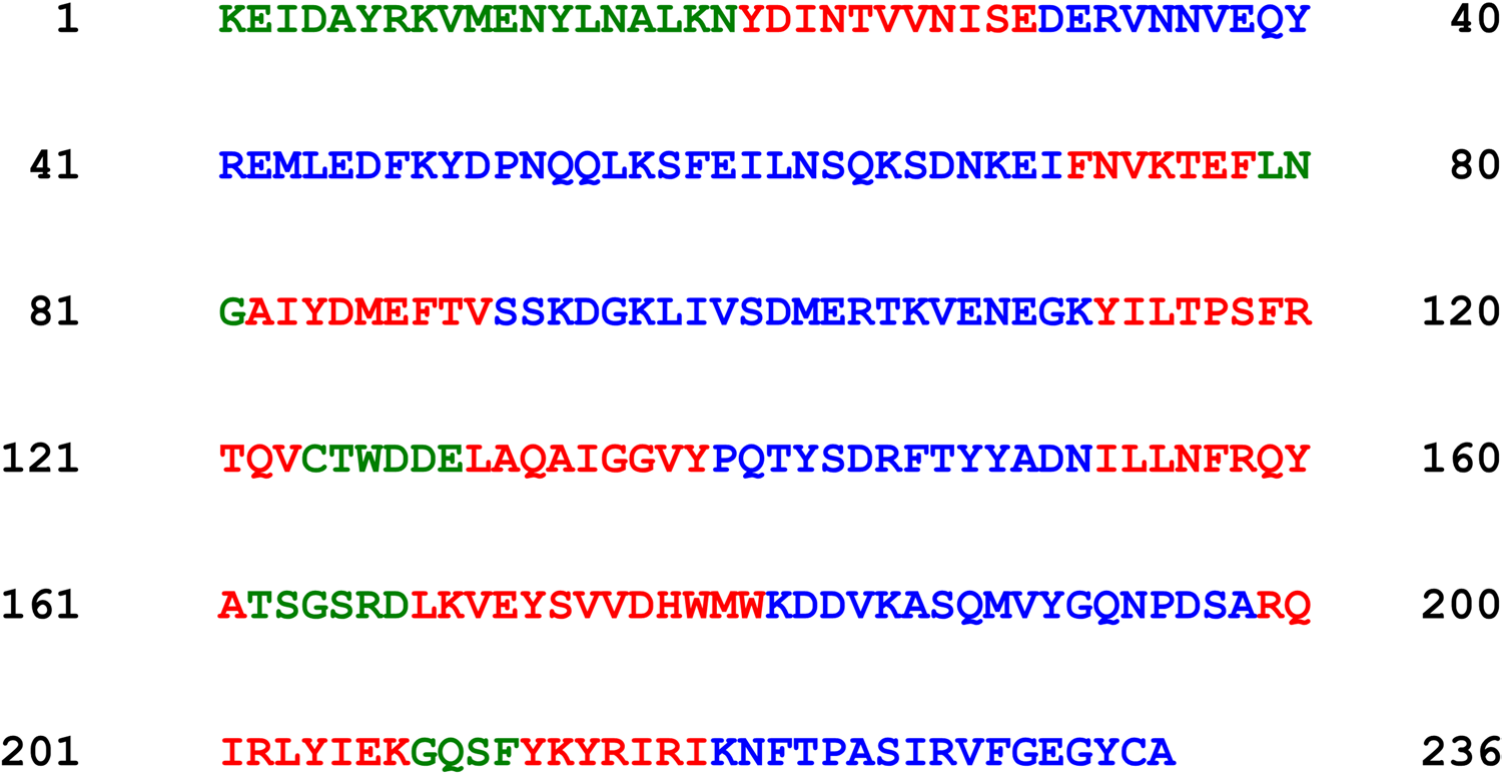
Analysis of the secondary structure of the mature form of cCPB2 (without signal sequence) for β-strands using PRED-TMBB (http://bioinformatics.biol.uoa.gr/PRED-TMBB/input.jsp). Amino acid stretches that could form transmembrane β-strands are colored in red. Stretches in blue or in green are exposed to different sides of the membrane to the aqueous phases. Some of the green stretches could form turns between two beta-strands

To get some insight in the putative channel structure we studied the secondary structure of cCPB2 using public domain programs (for example https://npsa-prabi.ibcp.fr/cgi-bin/secpred_consensus.pl). These programs suggested an only very small fraction of α-helical stretches of amino acids for both Beta2 toxins (cCPB2 and aCPB2) that are not sufficient to cross a membrane. This means presumably that they are not channel-forming proteins that have an α-helical backbone. The content of stretches with β-depleted sheets was much higher. Nevertheless, the search for transmembrane β-strands in both primary sequences with PRED-TMBB (http://bioinformatics.biol.uoa.gr/PRED-TMBB/input.jsp) did not classify both proteins as transmembrane proteins in terms of bacterial outer membrane proteins. This means presumably that only a small part of cCPB2, for instance one or two β-hairpins are involved in the channel similar as for other toxins, including CPB (Shatursky et al. 2000) as it is shown in Fig. 7 for the analysis of the mature cCPB2 protein with PRED-TMBB. In this figure, red colored stretches of amino acids could form β-strands in membranes. Water-soluble parts of the sequence are given in green and in blue. Taken together, this suggests that cCPB2 could be a member of (pore-forming toxins) PFT containing β-strands.

### Negative charges control ion transport through the consensus CPB2 channels

**Fig. 8:**
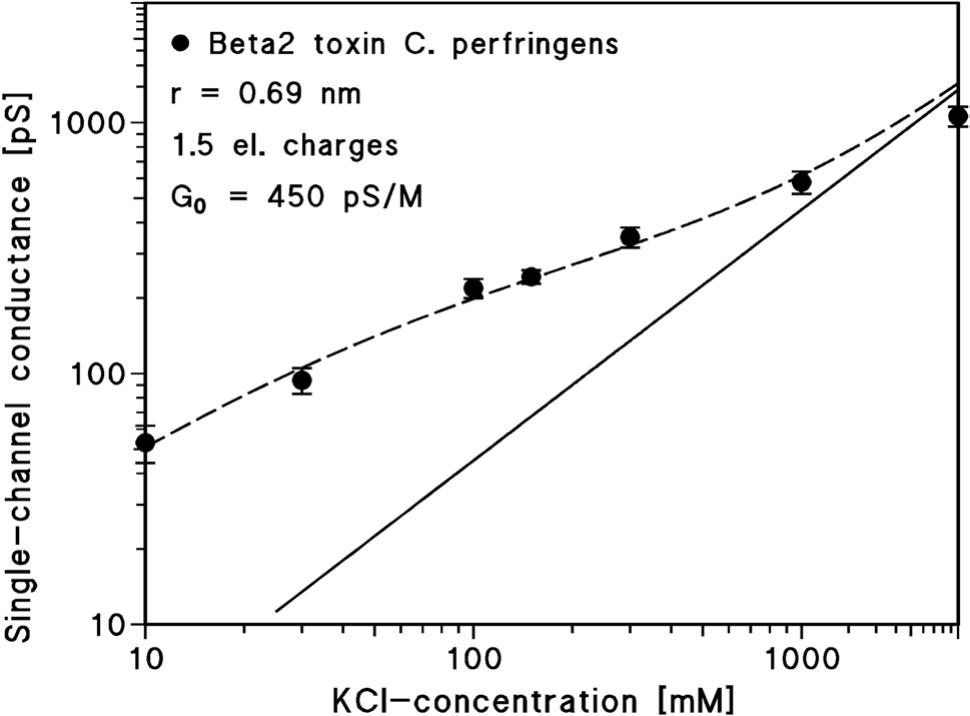
Single-channel conductance of the cCPB2 channel of *C. perfringens* as a function of the KCl concentration in the aqueous phase. The broken line represents the fit of the single-channel conductance data (filled circles ± SD, taken from Table 1) with Eqs. (1−4), using 1.5 negative point charges (*q* = − 2.4 × 10^–19^ As) and a channel diameter of about 1.4 nm as fit parameters. The straight line shows the single-channel conductance of consensus CPB2 that would be expected without point charges assuming a single-channel conductance of 450 pS/M. It corresponds to a linear graph between channel conductance and bulk aqueous. The number of single events used to calculate the average single-channel conductance (and SD) is given in Table 1, column 4

The analysis of the single-channel conductance and the selectivity of the CPB2 channels demonstrated that negative charges are involved in ion transport. A fit of the single-channel conductance as a function of the bulk aqueous concentration using Eqs. (1–4) allows some insight in the properties of the cCPB2 channel. Figure 8 shows the fit of the data using two parameters, the presumed radius r of the channel and the charge q. The latter number has to be taken as tentative because the dielectric constant of the material (lipid, protein, water) near the charge is largely unknown (Trias and Benz 1993). The size of the cCPB2 channel should on the other hand be more precise because it is controlled by the decay of the electrical potential in the aqueous phase, which should be given by the Debye length, also in an aqueous channel (Trias and Benz 1993). According to the fit, the diameter of the consensus CPB2 channel should be around 1.4 nm, which means that neutral or positively charged molecules with a molecular mass up to 600 Da could permeate the channel formed by cCPB2. A diameter of 1.4 nm is very similar to that of the CPE channel and to the diameters of the other toxin channels listed in Table 3 with the exception of that formed by the *C. septicum* alpha-toxin (Knapp et al. 2010). Heptamers form presumably all these channels by contributing seven β-hairpins to it.

**Table 3:** Comparison of the channel properties of cCPB2 with those of *C. perfringens* enterotoxin (CPE), aerolysin of *Aeromonas sobria*, alpha-toxin of *Staphylococcus aureus*, epsilon toxin of *C. perfringens* (ETX) and *Clostridium septicum* alpha-toxin

Toxin	Single-channel conductance in 1 M KCl	Zero-current membrane potential for fivefold KCl gradients	Permeability ratio *P*_K_/*P*_Cl_
	G [pS]	*V*_m_ [mV]	*P*_K_/*P*_Cl_
cCPB2	642	+ 33	11.8
CPE	623	+ 33	11.4
Aerolysin	650	− 24	0.21
*S. aureus* α-toxin	820	− 22	0.25
ETX	550	− 19	0.30
*C. septicum* alpha-toxin	1250	− 15	0.38

*V*_m_ is the electrical potential on the dilute side minus the potential of the concentrated side for a fivefold KCl-gradient across the membranes. The membranes were formed from diphytanoyl phosphatidylcholine/*n*-decane. The aqueous salt solutions were unbuffered and had a pH value of about 6. The permeability ratio, *P*_K_/*P*_Cl_, was calculated using the Goldman–Hodgkin–Katz equation (Benz et al. 1985). The results for epsilon toxin were taken from Petit et al. (2001) and those for aerolysin and α-toxin were taken from Chakraborty et al. (1990). The results for *C. septicum* alpha-toxin were taken from Knapp et al. (2010)

### Toxins/proteins related to cCBP2

**Fig. 9:**
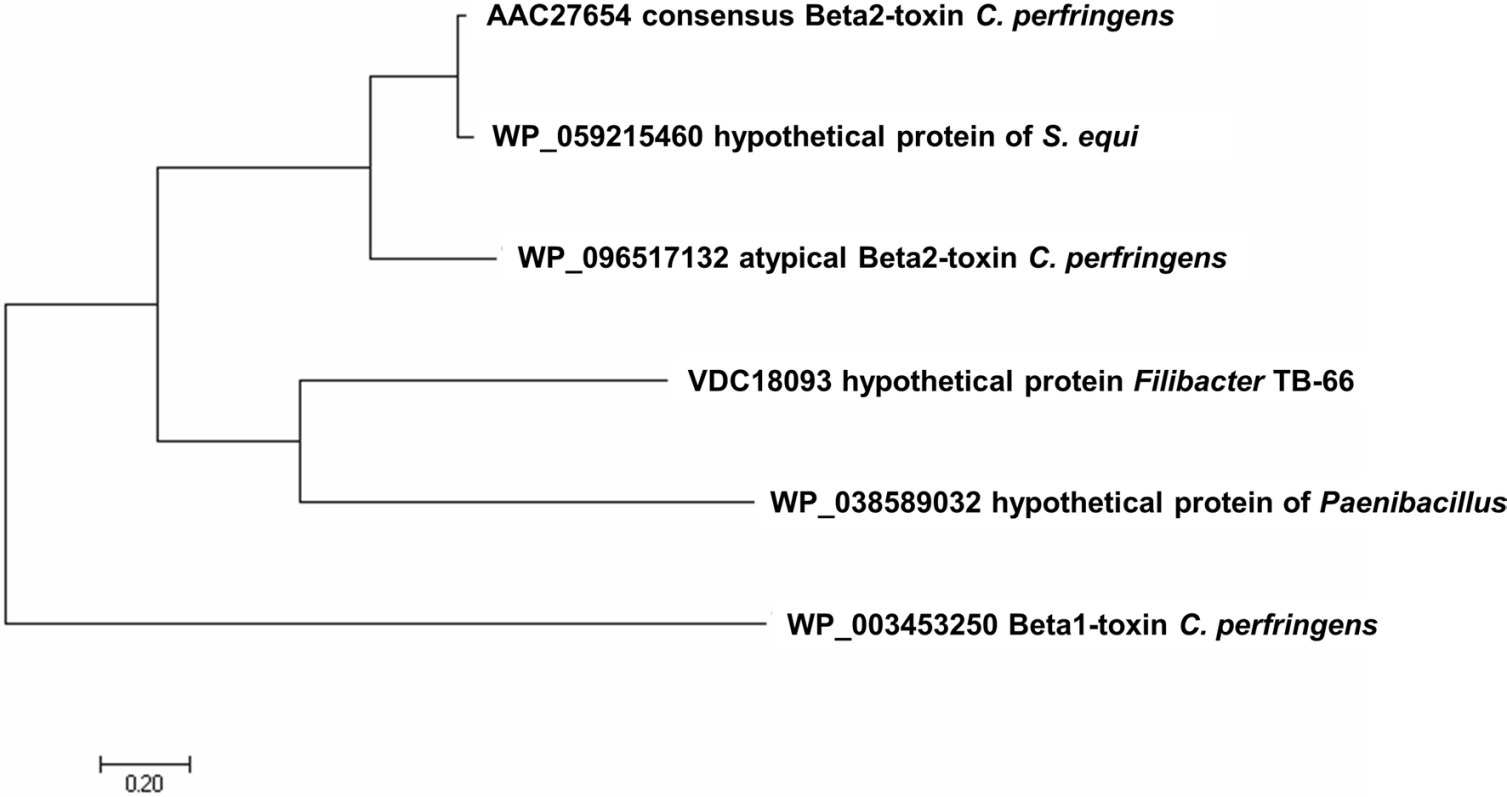
Cladogram representing the phylogenetic relationships of Beta2 toxin like proteins from different *Firmicutes*. The tree was generated using protein sequences downloaded from the NCBI protein database with the indicated identifiers. The multiple sequence alignment was calculated with Multiple Sequence Alignment (MUSCLE) (https://www.ebi.ac.uk/Tools/msa/muscle/). The tree was obtained using the program MEGA7 (Kumar et al. 2016)

We performed a BLAST search (https://blast.ncbi.nlm.nih.gov/Blast.cgi) for proteins that are related to cCPB2. We obtained four proteins that are in sequence and length related to cCPB2, which means presumably that they act also as toxins. The highest homology to cCPB2 has a hypothetical protein from *Streptococcus equi* (WP_059215460), where the mature protein is identical to the mature cCPB2. The next relative is a hypothetical protein FILTAT 026 (VDC 18093; 269 aa) from the *Firmicute* bacterium *Filibacter sp. TB-66*, which is with a similarity of only 20.77% already distantly related to cCPB2. An even smaller similarity to cCPB2 has a hypothetical protein (280 aa, WP_038589032.1) from the *Firmicute Paenibacillus* sp. FSL H7-0357, which has only a similarity of 18.3%. For these proteins, cCPB2, aCPB2 and CPB, we constructed a phylogenetic tree of the CPB2-like proteins from *Firmicutes* using the program MEGA7 (Kumar et al. 2016). The results are shown in Fig. 9.

There exist presumably more proteins from the phylum *Firmicutes* that could be related to cCPB2. However, when the relation is too distant, these proteins are no longer recognized by the BLAST search. The interesting point is in any case that the bacteria producing proteins related to CPB2 belong all to the phylum *Firmicutes*.

## Conclusion

Here, we show that cCPB2 is a PFT which forms cation-selective channels of about 1.4 nm diameter in lipid bilayers. The lack of predicted long alpha-helical structures in cCPB2 amino acid sequence suggests that cCPB2 does not belong to the family of toxins, where the channels are lined up by α-helices (α-PFTs). Figure 7 suggests that it is possible that the pore is lined up by β-strands (β-PFTs) because some amino acid stretches in the primary sequence of cCPB2 can serve as amphipathic transmembrane β-hairpins. This is supported by the fact that *C. perfringens* produces numerous beta-strand PFTs, which despite low amino acid sequence homology share conserved structures. No alpha-helical PFT has been identified in *C. perfringens* (Popoff 2014). Pore formation likely accounts for the biological activities of cCPB2.

## Data Availability

All the data are available in the manuscript.
